# Supplementary material for: Cycloastragenol Inhibits Experimental Abdominal Aortic Aneurysm Progression
Source: Biomedicines. 2022 Feb 2;10(2):359. doi: 10.3390/biomedicines10020359 (PMC8962318; doi:10.3390/biomedicines10020359)
Supplement: Supplementary file 1 [file biomedicines-10-00359-s001.zip › biomedicines-1546602-supplementary.pdf]

Supplementary Figure S1.

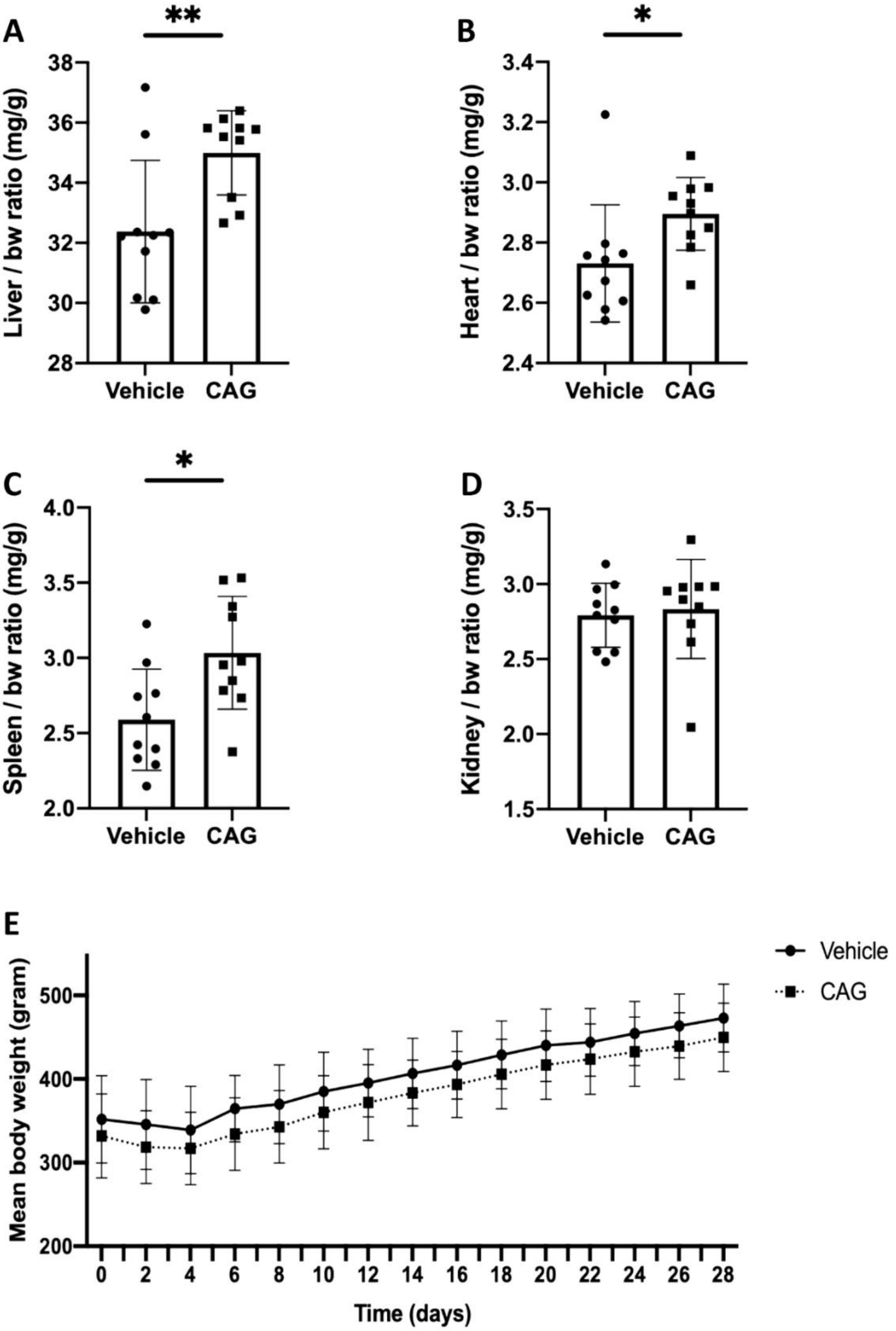

Supplementary Figure S1. Body weight changes and tissue weights during 28 days of CAG treatment. **A**) Mean body weight increase in CAG and vehicle group from day 0 prior to surgery, to day 28 at termination.. **B**) Liver to body weight ratio; (p = 0.0075), **C**) Heart to body weight ratio (p = 0.0358), **D**) Spleen to body weight ratio (p = 0.012), **E)** Kidney to body weight ratio (p = 0.744). Sample size: n = 10/10. Values are presented as mean ± standard deviation. * indicates $p < 0.05$, ** indicates $p < 0.001$.

Supplementary Figure S2

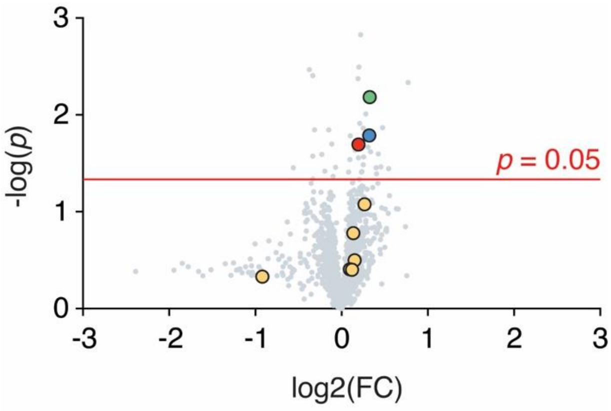

Supplementary Figure S2. Volcano plot showing changes in aneurysmal protein levels between CAG treatment and vehicle treated groups using proteomic analysis. Red line marks unadjusted *p*-value = 0.05. Red dot marks Fibulin-5, blue dot marks Aquaporin-1 and green dot marks Prostacyclin synthase. Yellow dots mark VSMC markers. Data is not adjusted for multiple testing (n = 10/10).

Supplementary Table S2:

CAG treatment does not affect aneurysm protein levels related to VSMC contractile related phenotype compared to vehicle treated rats

Protein focus	Accession	Description	Fold change	p-value
VSMC	Q63862	Myosin-11	1.20	0.084
VSMC	P62738	Actin, aortic smooth muscle	1.10	0.166
VSMC	P31232	Calponin-1	1.15	0.309
VSMC	Q08290	Myosin regulatory light polypeptide 9	1.07	0.390
VSMC	Q64122	Tropomyosin beta chain	0.53	0.468
VSMC	P58775	Transgelin/SM22	1.11	0.316

Supplementary Table S2. P*roteins related to vascular smooth muscle cells contractile phenotype is not de-regulated in CAG treated AAA tissue compared to vehicle treated AAA by explorative proteomics (n = 10/10). In this table, data is not adjusted for multiple testing.*
